# On Certain Eye Complications That Occur during and after an Attack of Gonorrhœa

**Published:** 1898-05-07

**Authors:** J. Astley Bloxam

**Affiliations:** Senior Surgeon to the Hospital


### Medical Progress and Hospital Clinics.

[*The Editor will be glad to receive offers of co-operation and contributions from members of the profession. All letters should be addressed to* The Editor, at the Office, 28 & 29, Southampton Street, Strand, London, W.C.]

### ON CERTAIN EYE COMPLICATIONS THAT OCCUR DURING AND AFTER AN ATTACK OF GONORRHŒA.

A Clinical Lecture given at Charing Cross Hospital, by J. Astley Bloxam, F.R.C.S., Senior Surgeon to the Hospital.

A case of disease of the eye due to gonorrhœa was shown, in reference to which, and to similar conditions, Mr. Bloxam made the following remarks: There are two conditions of disease of the eye due to gonorrhœa, namely—(1) A local contagion; (2) a constitutional condition due to absorption of the inflammatory products from the urethra. Condition 1 is of a rapid and painful character. In condition 2 the disease comes on insidiously, with nocturnal pain, and there is permanent impairment of the eye.

In cases like 1, which are due to a local contagion, the effect of inoculation with a minute particle of pus from the urethra is immediate, for the whole conjunctiva becomes of a brilliant red colour, like a piece of red flannel, and there is a swelling of the eyelids, which often become everted. The discharge is at first serous, and afterwards sanious, and if this discharge gets into the patient's other eye or into somebody else's eye it immediately becomes infected. There is effusion into the submucous tissue of the conjunctiva, and great swelling of the conjunctiva, and this effusion into the submucous tissue of the conjunctiva produces a condition called chemosis. Subsequently, the nutrition of the cornea becomes impaired, and sloughing of the cornea may result. The indication for treatment here is to relieve the pressure at the edge of the cornea. The acute affection of the eye just described may occur at any stage of gonorrhœa.

The immediate treatment of this condition when the case is seen early is that the eye must be constantly washed. The best salt with which to wash the eye at this stage is, said Mr. Bloxam, not alum or perchloride of mercury, as described in the text books, but a saturated solution of boracic acid, with which the eye should be washed every hour, night and day, and at the same time a little cocaine should be dropped in to relieve the smarting pain. The other eye should, of course, be carefully covered with a watch glass. If there is chemosis the conjunctiva must be incised all round the margin of the cornea, and the eyelids must be divided vertically and stitched back on to the forehead. This will relieve the pressure on the cornea, and will allow lotions to more freely come in contact with the conjunctiva. A plastic operation will afterwards be necessary to restore the normal shape of the eyelids. The conjunctiva should also be painted with a solution of nitrate of silver in water—argent. nit. gr. 2 to 4, ad. aq. fl. oz. 1. Immediately after the painting the conjunctiva should be washed with a solution of common salt in water. If instead of nitrate of silver a solution of perchloride of mercury 1 in 5,000 is used, one must shortly before apply a solution of cocaine, otherwise the perchloride of mercury will cause pain. If an ulcer of the cornea appears, the eye must be put at rest, and the only way to do this is by padding the upper lid in such a way as to steady the eyelid without producing pressure on the eyeball, for, as Mr. Bloxam demonstrated, very little pressure is sufficient to keep the eyelids at rest. The pad is made by taking a piece of cotton wool and making a pad as thick as two pennies, and no thicker; to avoid the pad of wool sticking against the eylids, place under it an oval piece of cambric or butter cloth, then over it place a bandage which must go round the forehead, and this bandage must be of the lightest possible character, for it is very important to keep an inflamed eye as cool as possible. The reason that pressure is put upon the eye is because, if the anterior layers of the cornea slough, the structures behind the cornea get pushed forward, and a conical cornea is likely to result.

The next thing to do is to relieve the congestion of the vessels going to the cornea, and the best method of doing this is by counter-irritation, of which the best form is the blister, which should be placed either in front of, or immediately behind, the ear. If, as often happens, two or three blisters are required, then place them first in front of, and afterwards behind, the ear. Next the congestion of the eye must be relieved by relieving the circulation, and the best way of doing this is by means of hot belladonna fomentations in the proportion of belladonna ʒi. to water a pint. In bathing an eye which is inflamed, care must be taken that the patient sits upright in a chair, and puts the sponge saturated with the lotion up to his eye, and that he does not bend over a basin, for, if he does, the blood from the back of the head runs downward into the eye, and makes the congestion worse.

To relieve the feeling of tension in the deep structures of the eyeball, 2 minims of a solution of atropine, in the strength of atropine 2 gr. to 4 gr., ad aq. fl. oz. 1, must be dropped into the eye three times a day. Quinine, or bark and ammonia must also be given, because these patients are in feeble health and require support. Do not shut them up in dark rooms, but send them out into the fresh air, and cheer them up, and let them have cheerful companions; for if they are shut up in dark rooms they mope and get run down in health, and then the ulcer does not heal.

In the second condition, which was referred to at the commencement of the lecture, where there is a constitutional condition due to absorption of the inflammatory products from the urethra, the state of affairs is more serious, because there is an inflammation of the deep structures of the eye—namely, of the choroid and sclerotic.

The first symptom is pain in the eyeball, and then on looking at the eye it is seen to be inflamed, but less so than in cases due to local contagion. There is photophobia. Now, if one looks carefully at the eye this condition may be observed: Immediately around the edge of the cornea there is a small pink zone, which consists of very fine congested blood-vessels. There is no chemosis, and the blood-vessels are beneath the conjunctiva, for the conjunctiva can be moved over them. This condition is characteristic of iritis and sclerotitis. It will be noticed, too, that the eye has lost its normal lustrous appearance, and that the pupil is irregular and does not react to light, and that there is exudation on the surface of the iris. This exudation comes on very insidiously, and if it is neglected adhesions of the iris will probably form.

The case which was shown was an example of the kind of disease just described. He was a man aged 27, who had gonorrhœa eight weeks previously. He had a shallow ulcer in the cornea, showing a small depression in the front layer. This was shown to be almost filled with a layer of opaque lymph, which opaque lymph would probably later on clear up, and the cornea would resume its normal transparency. Mr. Bloxam here remarked that lead or nitrate of silver lotions should never be used for this class of cases. The best application was boracic fomentation, a saturated solution. One point, too, should be remembered—namely, that one should never treat an inflamed eye without first testing the tension of each eye by means of the index finger of each hand lightly applied to the eyeball; for if there is increased tension there are changes in the vitreous and other structures of the eyeball—general ophthalmitis; and if these changes are present, there is danger of sympathetic ophthalmia of the sound eye.

Regarding the general treatment of these cases, hydrarg. perchlor. and pot. iodide should be given. Quinine in small doses twice a day is also useful. They usually recover with tremulous iris, or with duskiness or haziness of the vitreous, or with some form of altered or damaged eye. Keep up the general health. Leave the gonorrhœal discharge alone. The books recommend injections, but the lecturer's experience was never do anything to irritate the urethra, but simply wash the parts about the meatus and bathe them with warm boracic lotion in the same way that the eye is bathed. So do not give injections, but simply keep the discharge from the urethra as aseptic as possible. If there is pain give sandal oil. The patient's strength must also be kept up by good nourishing food; but the iodide and perchloride are the things with quinine.

Since this lecture was given the man has left the hospital; the ulcer has healed, and the condition of the iris and sclerotic has cleared up, although haziness of vision exists. This case is most interesting, for it raises the question, Could this man have had a urethral chancre, and could this condition, after all, be a sequela to syphilis? Such said the lecturer is not my opinion, for he presented no other symptoms which could be in the slightest way referred to syphilis; and this condition, following after gonorrhœa, I have frequently observed in many cases associated with rheumatism, or so-called rheumatic symptoms, which, however, in my opinion, are the result of septic absorption following on the condition of the urethra.

